# Evaluation of Fatigue Life of CRM-Reinforced SMA and Its Relationship to Dynamic Stiffness

**DOI:** 10.1155/2014/968075

**Published:** 2014-06-18

**Authors:** Nuha Salim Mashaan, Mohamed Rehan Karim, Mahrez Abdel Aziz, Mohd Rasdan Ibrahim, Herda Yati Katman, Suhana Koting

**Affiliations:** ^1^Center for Transportation Research, Civil Engineering Department, Faculty of Engineering, University of Malaya, 50603 Kuala Lumpur, Malaysia; ^2^Universiti Tenaga Nasional, Putrajaya Campus, Jalan Ikram-Uniten, 43000 Kajang, Selangor, Malaysia

## Abstract

Fatigue cracking is an essential problem of asphalt concrete that contributes to pavement damage. Although stone matrix asphalt (SMA) has significantly provided resistance to rutting failure, its resistance to fatigue failure is yet to be fully addressed. The aim of this study is to evaluate the effect of crumb rubber modifier (CRM) on stiffness and fatigue properties of SMA mixtures at optimum binder content, using four different modification levels, namely, 6%, 8%, 10%, and 12% CRM by weight of the bitumen. The testing undertaken on the asphalt mix comprises the dynamic stiffness (indirect tensile test), dynamic creep (repeated load creep), and fatigue test (indirect tensile fatigue test) at temperature of 25°C. The indirect tensile fatigue test was conducted at three different stress levels (200, 300, and 400 kPa). Experimental results indicate that CRM-reinforced SMA mixtures exhibit significantly higher fatigue life compared to the mixtures without CRM. Further, higher correlation coefficient was obtained between the fatigue life and resilient modulus as compared to permanent strain; thus resilient modulus might be a more reliable indicator in evaluating the fatigue life of asphalt mixture.

## 1. Introduction

The design of asphalt concrete mixture involves the selection and proportioning of materials to obtain the desired properties in the finished product. The main serious distresses associated with asphalt concrete pavement are fatigue cracking, which occurs at intermediate and low temperatures, and rutting, which occurs at high temperatures. These distresses reduce the service life of the pavement and increase the maintenance costs [1].

Fatigue is defined as a phenomenon by which the internal structure of material is gradually weakened by repeated applications of stresses lower than its ultimate failure stress. Moving loads that generate alternating compressive and tensile flexural stresses on both upper and lower surfaces can cause pavement's fatigue cracks and failures. It generally appears in the form of alligator or map cracking that is initially confined to localized zones and spreads at an increasing rate [2]. The resistance of asphalt mixtures to cracking is essentially dependent upon its tensile strength and extensibility characteristics. These can be achieved by simply increasing the bitumen content of the mix; however, such attempt may have an adverse effect on the mix stability. The use of softer bitumen can also improve the mix flexibility, but this may be achieved at the expense of the tensile strength and stability of the mix.

Adedimila and Kennedy [3] reported that lots of other factors could affect the fatigue life. They concluded that the fatigue life increases with the decrease of testing temperature. Flaky particles usually produce a significantly shorter fatigue life than the mixture that contains rounded particles and fatigue life increases with the decrease of stiffness. For that constant strain test, fatigue life increases with the increase of stiffness. The fatigue properties of bituminous mixtures are never considered in isolation from the stiffness of the mix, which determines the magnitude of the tensile strain experienced by the material and its life to crack propagation.

The proper measurements of the fatigue response of bituminous mixes would demand a laboratory test that involves the application of stress or strain level which is applied frequently until failure occurs, either by complete fracture or by a significant reduction in stiffness of material. The fatigue behaviour of the mix is identified by the relationship between the strain or stress level and the number of load repetitions until failure [2, 4].

Stone matrix asphalt (SMA) is a gap-graded asphalt mixture that has gained popularity worldwide. SMA was first developed in Germany during the mid-1960s to provide maximum resistance to rutting caused by the studded tyres on road [5]. Earlier in the 1990s, SMA technology was widely used in the United States; however, most researchers' reports highlighted the mixtures' great possibility in rutting resistance but ignored any potential fatigue resistance of SMA [6]. Due to the nature of SMA mixes (gap-graded) and the relatively large proportion of asphalt content, stabilisation is required to inhibit drain down of bitumen. These requirements can be achieved by adding fibre or polymer modifier, since commercial polymer is not economical in terms of usage [2]; therefore using waste materials such as CRM in the asphalt mixture has been found to be more economical and environment-friendly [7–10]. The use of crumb rubber modified bitumen binder seems to enhance the fatigue resistance, as illustrated in a number of studies [11–14].

Since there are only a limited number of studies that investigate the fatigue life of SMA mixtures using bitumen modified with waste materials, thus the main aim of the current study is to investigate the fatigue properties of SMA mixtures reinforced with waste tyre rubber. In this paper, studies conducted on factors affecting fatigue life of asphalt pavement have been taken into consideration.

## 2. Factors Affecting Fatigue Life of Asphalt Pavement

Road pavements are subjected to a repeated passage of wheel loading of varying magnitude and intensity that could induce fatigue failure by cracking and deformation as a result of fluctuating stresses and strains within the pavement layers. Cracks generally initiate at the bottom of pavement layers where tensile strain is highest and then propagate towards the surface. Such cracks weaken the ability of the pavement structure to support load. The fatigue behavior of bituminous materials is known to be affected by testing mode, loading variables, and mixture variables.

### 2.1. Testing Mode

The testing mode describes the variation of the stress and strain levels during the fatigue loading test. Fatigue tests on bituminous specimens are performed under either controlled stress (load) or controlled strain (displacement). The laboratory fatigue life of the bituminous materials depends greatly on which mode of testing is used. In the controlled stress fatigue testing, the applied stress is kept constant and the strain gradually increases as the specimen is weakened. Accumulations of strains culminate into complete fracture of the specimen that occurs almost immediately after crack initiation. The controlled strain testing mode involves the application of a constant strain (displacement). As testing progresses, the specimen gets weaker due to accumulated damage. The stress (load) required to produce or maintain the initial strain gradually decreases. According to Roberts et al. [15], the constant stress conditions are applicable to thick bituminous pavement layers in excess of 130 mm whereas thin pavement layers should be tested in constant strain mode. The controlled strain mode of testing displays better fatigue life than the controlled stress mode of loading if all other variables are kept constant [16].

### 2.2. Loading Variables

The loading variables affecting fatigue life are loading waveform, rate of loading (frequency), and rest period. Raithby and Ramshaw [17] are of the opinion that the influence of loading waveform is sufficiently small in laboratory fatigue testing of bituminous specimens. They found that the square waveform resulted in the shortest fatigue life and the longest triangular waveform. Loading frequency and duration have a significant effect on the fatigue life. The effect of loading frequency is mainly accounted for by its effect on the stiffness of the material. Higher frequencies of loading cause higher mix stiffness. In other words, increasing the frequency of the load pulse increases the fatigue life. Decreasing the duration of load pulse (increasing the rest period) also increases the fatigue life (increase in stiffness) [18]. Raithby and Sterling [19] found that increasing the load repetition from 30 to 100 applications per minute reduced fatigue life by 20%. The application of a continuous mode of loading in fatigue tests does not resemble the kind of loading experience by the road pavement. In practice, the pavement is loaded discontinuously and there is a rest period between successive wheel loads of a vehicle or between successive vehicles. The introduction of rest period into a laboratory fatigue testing program attempts to simulate the loading pattern on a pavement structure under actual traffic conditions. A rest period is the time between consecutive applications of wheel loads. The beneficial effect of rest periods on prolonging fatigue life has been confirmed by many investigations [20–22]. The inclusion of a rest period can give an increase in fatigue life of between 5 and 25 times depending on the ratio of the loading duration to the rest period [21]. Rest periods have been found to allow time for the cracks in the bituminous samples to heal and the stresses and strains to relax due to the viscous flow of the bitumen. Rest periods are different for different roads and for different times of the day as they are dependent upon the volume of traffic. For instance, in one transportation research investigation, a mere one-second rest period increased the number of cycles to failure by a factor of 25 when compared with the fatigue life under continuous sinusoidal loading. However, there is a limit to which the rest period can be increased, above which an additional increase in rest period does not influence the fatigue life. This limit was found to be dependent on test temperature [19, 21]. It is unlikely that the predicted number of load applications to failure obtained from a laboratory study will compare very well with actual numbers obtained from the field due to differences in loading conditions and testing environment. A direct comparison between observed pavement fatigue life and that indicated by the laboratory fatigue curves was found to give erroneous results [21]. In the field, the load applications vary widely. A study [2] suggested that the fatigue life in the field is often as much as 20 times greater than the fatigue life of specimens measured in the laboratory. The effect of rest period was thought to have the greatest effect on increasing the fatigue life of actual road pavements.

### 2.3. Mixture Variables

The composition of a bituminous mixture directly influences fatigue response of a bituminous mixture. The more important mixture variables that have been quoted in the literature [23] include mix stiffness, bitumen content and properties, porosity and degree of compaction, and aggregate characteristics.

#### 2.3.1. Mix Stiffness

The stress and strain are affected by both temperature and time of loading. They are an important material characteristic in fatigue since the stresses and strains resulting from traffic loading moving at different speeds and those which arise when the pavement is subjected to different temperature during loading are dependent on stiffness. In the stress controlled mode of loading, mixtures with a high stiffness exhibit an increase in fatigue life; conversely in the strain mode of loading there is a decrease in the fatigue life. Read and Collop [18] showed that, for crack propagation relationships, lower stiffness pavements have longer service life than high stiffness ones. Fatigue life is reduced by the use of harder bitumen [24].

#### 2.3.2. Bitumen Content and Properties

Bitumen content affects the fatigue response of bituminous mixtures. Increasing the bitumen content increases the fatigue resistance of a mix. Goddard et al. [25] have shown that a one-percent reduction in bitumen content can reduce the fatigue life by 70%. For the longest fatigue life, the volume of bitumen should be as high as possible, but this is limited according to permanent deformation requirements [18]. The effect of the type and hardness of bitumen on the fatigue characteristics of bituminous materials can be accounted for by their effect on mix stiffness. Generally, in the controlled stress mode of loading, as the hardness of the bitumen is increased, thus the mixtures stiffness is increased resulting in a higher fatigue life at a particular stress level [26]. Fatigue response is dependent on the mode of loading and the stiffness of the mixture. In general, factors tending to increase mix stiffness tend to increase the service life at a particular level of stress. In the stress controlled mode of loading, fatigue behavior of bituminous mixture can be enhanced by increasing the bitumen content, using more viscous bitumen and achieving a well-compacted mixture. The converse is true for the strain controlled mode of loading.

#### 2.3.3. Air Void and Compaction

The influence of compaction level on the fatigue life indicated that the lower the volume of air voids the higher the resultant fatigue life. Barksdale [16] reported an increase in fatigue life by a factor of 9 when the porosity is reduced from 8 to 6% and by a factor of 200 when the porosity is reduced from 6 to 3%. Epps and Monismith [26] have also shown that the fatigue life of asphaltic concrete is reduced from 10 to 30 percent for each 1-percent increase over normal air void. However, there is a compromise between fatigue and permanent deformation. If the air voids are reduced by too much, they will become overfilled with bitumen, pushing the aggregates apart, and the mixture will rut very quickly [18].

#### 2.3.4. Aggregate Characteristics

Goddard et al. [25] showed that aggregates (type and gradation), filler content, and compaction level have little influence on the laboratory fatigue life of bituminous specimens; their effect is mainly accounted for by their effect on the dynamic stiffness of the mix. Angular aggregate increased fatigue life by a factor of 1.14 relatively to round aggregates [24]. Read and Collop [18] reported that the shape of the aggregates plays a major role in the crack propagation phase with flaky aggregates oriented normally to the applied load giving slower rate of crack propagation than that obtained using normally spherical aggregates. In addition, the strength and toughness of the coarse aggregate particles play an important role in the fatigue failure of mixture with rough textured aggregates, whereas the interface bond strength controls the fatigue failure of the mixtures with polished aggregates. Recent study [27] investigated the effects of aggregate size, mixing temperature, and asphalt content on fatigue failure. Indirect tensile stiffness modulus and indirect tensile fatigue test were conducted. Results showed that fatigue life decreased with increase of the temperature. Also, hot mix asphalt had greater fatigue life compared to stone mastic asphalt. It referred to dense grade inherent structure which interlocked better to each other in comparison with stone mastic asphalt [27]. Further, it was concluded that increasing the asphalt content will make the mixture less stiff and, therefore, cause less fatigue resistance; however, the effect of aggregate gradation on fatigue life is more considerable than the effect of asphalt content.

## 3. Materials and Methodology

### 3.1. Materials

A bituminous binder of 80/100 penetration grade was used. Tables 1 and 2 show the characteristics of the bitumen used in this research. The crushed granite aggregates used throughout the study were supplied by the Kajang quarry, a suburb near Kuala Lumpur, the capital city of Malaysia. The aggregate gradation of SMA 20 was selected according to Malaysian Standard Specification for Road Works (JKR) as illustrated in Table 3. Because of the importance of aggregate quality in SMA mixtures, several tests were done on coarse and fine aggregate particles, and the results are listed in Table 4.

In this study, the gradation of crumb rubber number 40 (0.45 mm) was selected. The density of crumb rubber is about 1.15 (gm/cm³). The crumb rubber modifier (CRM) produced by mechanical shredding at ambient temperature was obtained from Rubberplas Sdn. Bhd. (Malaysian supplier) with different percentages of 6, 8, 10, and 12% by weight of the binder. The elastomeric compositions for crumb rubber are natural rubber 30%, styrene-butadiene rubber (SBR) 40%, and butadiene rubber 30%. The physical properties for crumb rubber are presented in Table 5, and CRM chemical components are illustrated in Table 6. Ordinary Portland Cement (OPC) was added to the combined aggregate for bituminous mixture to serve as an adhesion and antistripping agent [2]. This study used 8% filler, 2% of which was OPC and 6% consisted of stone dust.

### 3.2. Samples Fabrications

To incorporate rubber in bituminous mix, CRM is blended with the aggregate before adding the binder to the mixture. The optimum binder content according to the Marshall method (ASTM D 1559) was chosen based on examining volumetric properties of the specimens as well as their stability and flow test results. The specimens were prepared at optimum asphalt content (OBC) using Marshall method and Table 7 illustrates Marshall characteristics at OBC. Five various amounts of OBCs have been obtained for five various CRM contents: 5.40%, 6.35% 6.45%, 6.50%, and 6.70% of OBC each for 0%, 6%, 8%, 10%, and 12% (all by weight of aggregate particles) of CRM content, respectively. For preparing SMA mixtures, 1100 g of mixed aggregate was placed in the oven at 160°C for 1.5 h. Bitumen was also heated at 120°C before mixing with aggregate particles. As the dry process method is utilised, crumb rubber modifier was added directly to the mixture. Mixing temperature was kept constant at the temperature between 160 and 165°C. The mixture was transferred into a Marshall mould. All samples were subjected to 50 blows of compaction by Marshall Hammer on each side of specimen at temperature of 150°C.

### 3.3. Test Methods

#### 3.3.1. Indirect Tensile Modulus Test

**Figure 1:**
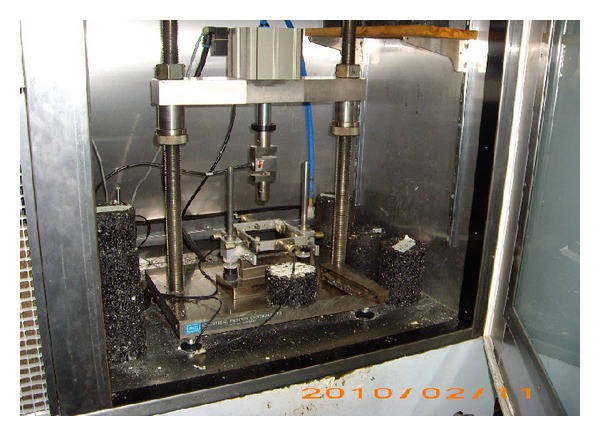
The testing machine (Universal Materials Testing Apparatus (UMATTA)) and the CR-reinforced SMA samples.

The indirect tensile test has emerged as the most convenient tool for measuring the stiffness modulus of bituminous mixtures in the laboratory. This test is carried out under repeated loading at low stresses so that the response of the specimen tested remains elastic. The test is regarded as nondestructive test conducted using UMATTA machine according to ASTM D4123 (as shown in Figure 1). The equipment is computer controlled and uses a pneumatic actuator to apply the load. Prior to testing, the specimen to be tested was left in the chamber at the prescribed testing temperature for a minimum of 4 hours. The test was performed at a controlled temperature of 25°C; the specimen is tested at four points of loading each perpendicular to one another. The stiffness modulus of the materials is calculated using the following relationship:

(1) $$\begin{array}{c} M_{R}=\frac{P\left(v+0.25\right)}{tH}, \end{array}$$

where *M*_*R*_ is the resilient modulus (MPa); *P* is the repeated peak load (*N*); *v* is Poisson's ratio; *t* is specimen thickness (mm); and *H* is total recoverable horizontal deformation (mm).

#### 3.3.2. Dynamic Creep Test

The creep test, using either one cycle load/unload (static creep) or cyclic loading (dynamic creep), is capable of providing much information concerning the materials' response characteristics. The interpretation of the strain/time response of materials undergoing a creep test provides significant parameters, which describe the instantaneous elastic/plastic and viscoelastic/plastic components of the materials response [2]. The test was conducted at temperature of 40°C for a period of 1 hour with loading stress of 100 kPa; the pulse period, pulse width, terminal pulse, and conditioning stress count were at 2000 ms, 200 ms, 1800 counts, and 1 kPa, respectively, by using the UMATTA apparatus [2].

#### 3.3.3. Indirect Tensile Fatigue Test

Universal Materials Testing Apparatus (UMATTA) was used to determine the repeated load indirect tensile test as a method of assessing the fatigue resistance of bituminous materials. Fatigue tests on bituminous specimens are performed under either controlled stress (load) or controlled strain (displacement). In the controlled stress fatigue testing, the applied stress is kept constant and the strain gradually increases as the specimen is weakened. The controlled strain testing mode involves the application of a constant strain (displacement) which is kept constant. In this study, indirect tensile fatigue test (ITFT) was carried out in controlled stress mode according to EN 12697.

The placement of the sample and test setup are similar to the resilient modulus test where the same loading jig is used. The specimen was exposed to repeated compressive loads with a load signal through the vertical diametrical plane. This loading developed a relatively uniform tensile stress perpendicular to the direction of the applied load, which induced permanent deformation leading to failure of the sample by splitting along the central part of the vertical diameter. The resulting deformation was measured and an assumed Poisson's ratio was used to calculate the tensile strain at the centre of the sample. During the test, the load and horizontal deformation were monitored continually and recorded at the preselected intervals using computer data system. The test was stopped when an obvious cracking appeared on the vertical axis. In this research study, three cyclic loading forces were used (200, 250, and 300 kPa), respectively. Loading cycle width was 100 ms; load cycle repeated time was 500 ms; and temperature was 25°C with axial displacement of about 5-6 mm.

The fatigue life is defined as the number of load cycles applied (cycles) resulting in either disintegration or a permanent vertical deformation. A stiffness reduction of 50% was used to present the sample failure due to fatigue deformation. Horizontal tensile strain also can be obtained as the function of stress and stiffness of mixture.

Raad and Saboundjian [11] investigated the fatigue life of asphalt concrete mixtures using the indirect tension fatigue test. During the indirect tension fatigue, the horizontal deformation was recorded as a function of load cycle. The test specimen was subjected to different levels of stress, for a regression analysis on a range of values. This allowed the development of the fatigue relationship between the number of cycles at failure (*N*_*F*_) and initial tensile strain (*ε*_*t*_) on a log-log relationship. Fatigue life (*N*_*f*_) of a specimen is number of cycles to failure for asphalt concrete mixtures. These models are created based on the relationship existing between stress, or strain, and fatigue life as below:

(2) $$\begin{array}{c} N_{f}=A{\left(\frac{1}{\sigma }\right)}^{n},\\ N_{f}=a{\left(\frac{1}{ɛ}\right)}^{b}, \end{array}$$

where *N*_*f*_ is the number of load cycles to failure, *σ* is applied stress, *ɛ* is initial strain, and *A*, *n*, *a*, and *b* are regression coefficients (fatigue parameters) which are related to mixture properties. Horizontal tensile strain also can be obtained as a function of stress and stiffness of mixture by using

(3) $$\begin{array}{c} {ɛ}_{x}\left(\max\right)=\frac{\left(\sigma_{x}\max\right)\left(1+3v\right)}{S_{m}}, \end{array}$$

where *ɛ*_*x*_(max⁡) is the maximum tensile strain at the center of specimen, *σ*_*x*_(max⁡) is the maximum tensile stress at the center of specimen, *S*_*m*_ is the stiffness modulus of specimen, and *v* is Poisson's ratio.

## 4. Results and Discussion

### 4.1. Indirect Tensile Test Results

**Figure 2:**
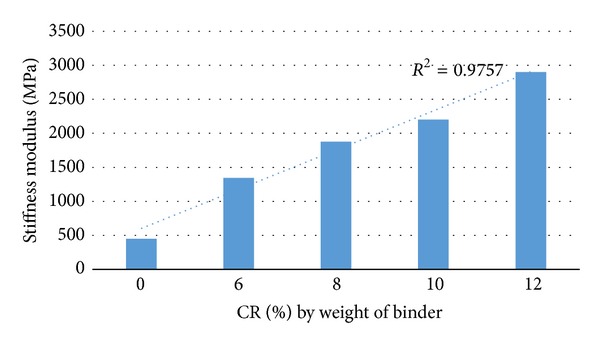
Stiffness modulus versus CR content at 25°C.

The tensile properties of asphalt mixtures are of interest to pavement engineers because of the problems associated with cracking. The resistance of asphalt mixtures to fatigue cracking is dependent upon its tensile properties, notably its tensile strength and stiffness.  Figure 2 shows the stiffness modulus (*M*_*r*_) variation against bitumen content for asphalt mixtures reinforced with different contents of CRM and nonreinforced asphalt mixture (containing 0% CRM). This means that at optimum conditions the control sample has a bigger elastic deformation than the rubberised samples under dynamic traffic loading conditions. As revealed in Figure 1, there is a marked variation between the reinforced and nonreinforced samples in the stiffness modulus (*M*_*r*_). As the crumb rubber content is increased, more bitumen is absorbed, which in turn increases the optimum binder content of the mix. It is evident that the stiffness modulus of reinforced asphalt samples is higher compared to the nonreinforced samples; however, the results of this research agree with the findings of previous studies [7, 9, 28]. Mixes with higher stiffness suggest that, apart from being stiffer, they are more resistant to deformation [1, 2]. IDT results (stiffness modulus) indicate that the increase in CRM content produces an improvement in the elastic properties of the studied mixtures. Modified bitumen improves the resilient modulus of asphalt mixtures compared to the control mixtures, due to higher viscosity and thick bitumen films leading to better resilience properties. Thus modified bitumen produces asphalt concrete mixtures with improved stiffness and subsequently higher load bearing capacity.

### 4.2. Dynamic Creep Test Results

**Figure 3:**
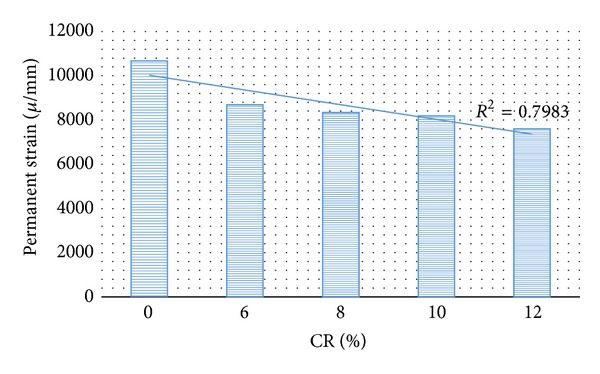
Permanent strain versus CR content at 25°C.

This test simulates the passage of moving traffic loads on the pavement to study the permanent deformation characteristics of bituminous materials and its ability to resist the creep distress under repeated load. The results obtained for dynamic creep test represented by the permanent strain for mixes at OBC with different CRM content at testing temperature of 25°C are given in Figure 3.

As shown in Figure 3, it is expected that with further inclusion of CRM and beyond certain CRM content the strain value of the mixture will increase higher than that of the nonreinforced mix causing a detrimental effect to the reinforced mix by reducing its resistance to permanent deformation. According to a study attributed to the effect of rubber content, enhancing the rubber content led to the increase in carbon black reacting with the natural rubber, which corresponded to the elastic part of the crumb rubber chemistry. It seems that higher CRM content has significant effect on the elastic recovery of the modified bitumen by increasing the rubber mass due to the absorption of maltenes from the bitumen binder. Thus, the modified binder became more elastic and thus improved its resistance to elastic deformation under high tensile stress [1, 9].

### 4.3. Indirect Tensile Fatigue Test Results

**Figure 4:**
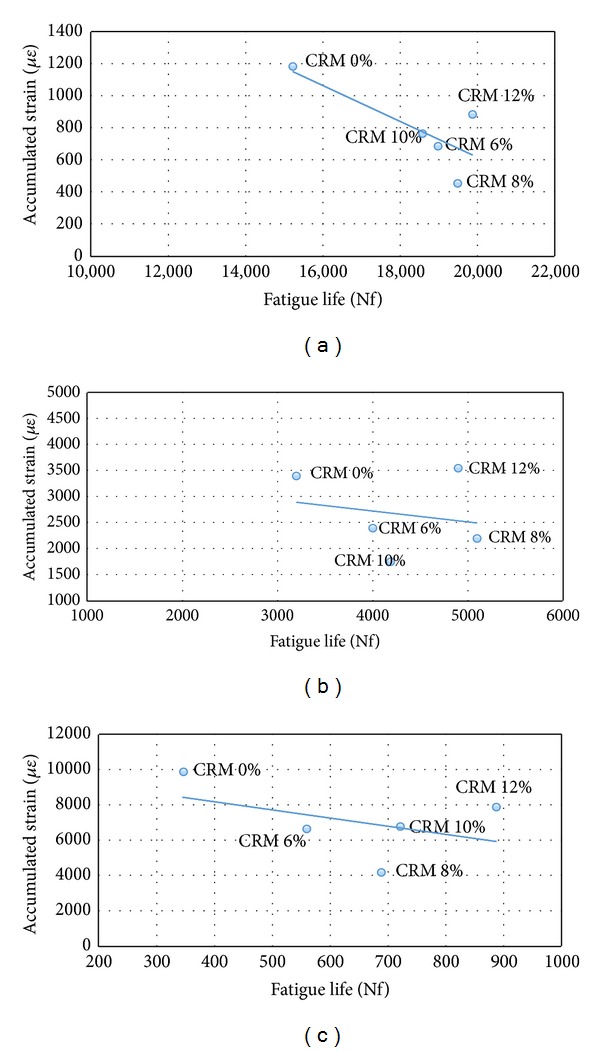
Fatigue life versus accumulative strain at different stress levels: (a) 200 kPa, (b) 300 kPa, and (c) 400 kPa.

The fatigue characteristics relating to the accumulated strain with the number of cycles to failure for the SMA mixes with and without CRM reinforcement are presented in Table 8 and Figure 4 for various stresses with fatigue predicting model. Table 8 and Figure 4 indicate that the addition of CRM binder into SMA mixture improved the fatigue life and reduced the accumulated strain. SMA mixture reinforced with 12% CRM resulted in high fatigue life and hence lower strain value. Also, it appears that the higher the stress is, the lower the fatigue life is. The basic fatigue life model confirms the aforementioned effects of crumb rubber content and stress levels on fatigue life. By looking at the fatigue model coefficients, some guidance may be provided. As the loading cycles are increased, the rate of tensile strain generation for both reinforced and nonreinforced specimens is found to be different. Crumb rubber modifier (CRM) leads to sustenance of higher tensile strains in asphalt samples. The high elasticity and tensile strength of crumb rubber allow asphalt samples to deter creep-caused cracks as well as reduce the generation and propagation rate of microcracks. The high tensile strength evident in CRM can deter crack generation and the propagation of microcracks in asphalt samples [1, 28].

The low content of CR (6, 8, and 10) has displayed insignificant effect on improving the fatigue life. As the crumb rubber content is increased, more bitumen is absorbed, which in turn increases the optimum binder content of the mix. It is observed that deviation from the optimum CRM (12%) content decreases the fatigue life of reinforced asphalt samples. The CRM asphalt deters tensile and vertical cracks from being effortlessly formed by horizontal tensile stresses and stops them from propagating. It seems that SMA mixtures tend to have lower fatigue lives at higher stress levels. This is probably due to chopped crumb rubber that is well distributed in bituminous matrix that highly resists the shear displacement and firmly prevents aggregate particles from any movement, thus increasing fatigue life by efficiently delaying crack propagation once the crack had been initiated [1, 4, 9].

## 5. Relationship between Fatigue Deformation, Stiffness Modulus, and Creep Strain of Reinforced SMA

Considerable effort and cost are required to measure the fatigue response of asphalt mixture using conventional laboratory procedure and to use the information so obtained to design fatigue resistance mixtures. The aim of these alternative procedures is to simplify both testing and analysis procedure to estimate the fatigue performance. Considered here in this section is an attempt to relate some of the conventional mix tests results (dynamic creep and resilient modulus) to fatigue response.

**Figure 5:**
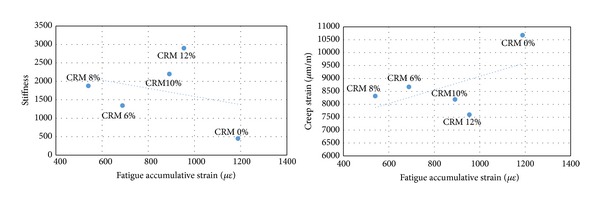
Fatigue versus stiffness and creep at 200 kPa stresses.

**Figure 6:**
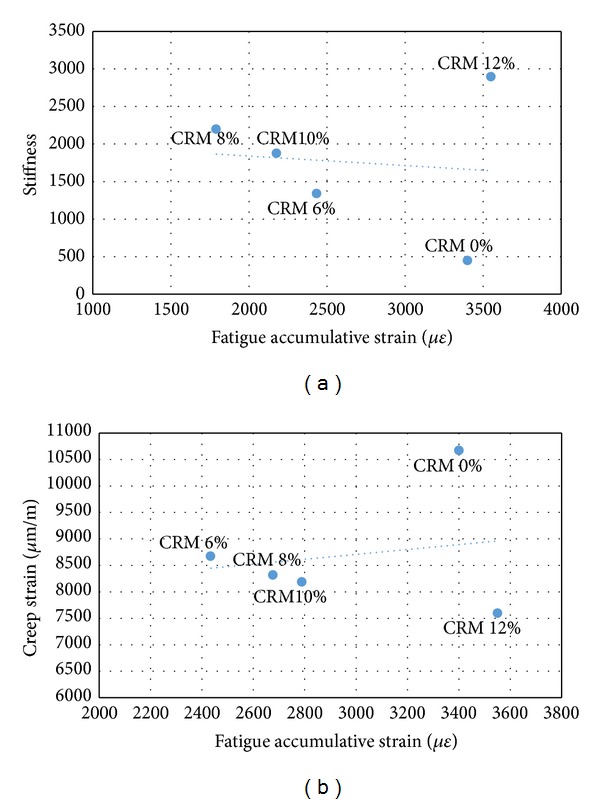
Fatigue versus (a) stiffness and (b) creep at 300 kPa stresses.

**Figure 7:**
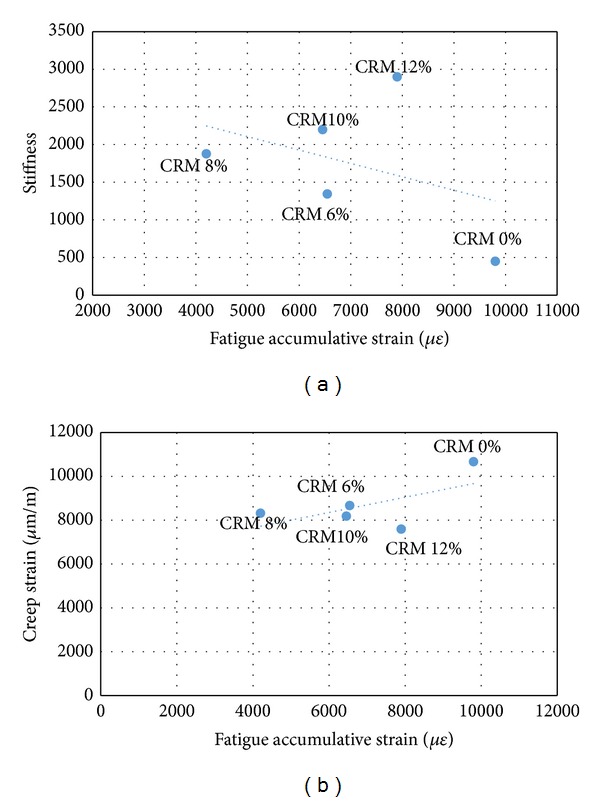
Fatigue versus (a) stiffness and (b) creep at 400 kPa stresses.

Figures 5, 6, and 7 show fatigue accumulated strain versus creep permanent strain and stiffness modulus, respectively, for the stress of 200, 300, and 400 kPa used in this study. The figures showed that the fatigue accumulated strain of the specimen resulted in a proportional correlation with the creep permanent deformation of the mixes, whereas an inverse correlation was obtained between the fatigue and stiffness properties. In simple words the results indicate that the decrease in fatigue accumulated strain due to the addition of CRM resulted in a decrease of creep strain of the specimen and in an increase in the stiffness properties.  Table 9 shows a strong correlation coefficient between the fatigue strain and stiffness about *R* ^2^ = 0.95, 0.89, and 0.93 for stress of 200, 300, and 400 kPa, respectively, while showing a lower correlation coefficient between the fatigue strain and creep strain about *R* ^2^ = 0.71, 0.67, and 0.68 for 200, 300, and 400 kPa stresses.

Even though fatigue test is considered a destructive test, it seems to correlate better with resilient modulus test which is nondestructive test as compared to the creep test which is destructive test. Resilient modulus test being nondestructive test is in fact a good indicator of mixture cohesion; on the other hand, creep test being a destructive test seems to account better for differences in internal friction among asphalt mixture than mixture cohesion. Therefore, resilient modulus test might be a more reliable test in evaluating the fatigue performance of asphalt mixtures as compared to dynamic creep test, or simply the significance of mixture cohesion in contributing to the fatigue resistance of asphalt is more important than mixture internal friction.

## 6. Conclusion

Highway pavements are subjected to a repeated passage of wheel loading of varying magnitude and intensity that could induce fatigue failure by cracking and deformation as a result of fluctuating stresses and strains within the pavement layers. Fatigue cracking is an important cause of road failure at low temperatures, but it has not been significant at elevated temperatures where permanent deformation is the predominant mode of failure. This resistance of bituminous mixtures to cracking is essentially dependent upon its tensile strength and extensibility characteristics. These can be achieved by simply increasing the bitumen content of the mix [1, 7].

Based on the study conducted, the following conclusions may be derived.

1. The dynamic stiffness (resilient modulus) of reinforced SMA samples containing various contents of CRM is significantly high in comparison with that of nonreinforced samples. This increased stiffness modulus, however, is not related to increased brittleness of reinforced asphalt samples. The stiffness modulus of reinforced samples is in fact less severely affected by the increased temperature compared to the nonreinforced samples.
2. The fatigue life of SMA reinforced samples is significantly improved with the use of CRM. The resistance of waste tyre rubber to the generated horizontal tensile stresses decreases the formation of vertical cracks and prevents these cracks from propagating along the diameters of asphalt samples. This in turn improves the fatigue life of reinforced samples. The relationships obtained are rational; the higher the stress level is, the lower the fatigue life is and the higher would be the accumulative strain.
3. It was obvious that the CRM content of 12% by weight of the total mix resulted in the highest performance in terms of stiffness and resistance to permanent deformation as compared to the ordinary mix.
4. Even though fatigue test is considered a destructive test, it seems to correlate better with resilient modulus test which is nondestructive test as compared to the creep test which is destructive test.
5. Higher correlation coefficient was obtained between the fatigue life and resilient modulus as compared to permanent strain; therefore, dynamic stiffness seems to be more adequate in evaluating the fatigue life of asphalt.

## Figures and Tables

**Table 1:** Properties of base binder grade 80/100 penetration.

Test properties	Value	Standard test
Viscosity at 135°C (Pas)	0.65	ASTM D4402
G∗/sin⁡⁡*δ* at 64°C (kPa)	1.35	ASTM D4-P246
Ductility at 25°C	>100	ASTM D113
Softening point at 25°C	47	ASTM D36
Penetration at 25°C	88	ASTM D5
Flash point (°C)	300	ASTM D9
Fire point °C	317	ASTM D9
RTFOT aged G∗/sin⁡⁡*δ* at 64°C (kPa)	6.022	ASTM D2872
PAV aged G*sin⁡⁡*δ* at 25°C (kPa)	3122.5	ASTM D6521

**Table 2:** Chemical composition of bitumen (%)(Standard IP 469).

Bitumen	80/100
Saturates	5.4
Aromatics	72.5
Resin	15.5
Asphaltenes	6.6

**Table 3:** SMA 20 aggregate gradation [4].

BS sieve	Min.	%Passing		%Retained	Weight (G)
		Max.	Mid.		
19	100	100	100	0	0
12.5	85	95	90	10	110
9.5	65	75	70	20	220
4.75	20	28	24	46	506
2.36	16	24	20	4	44
0.6	12	16	14	6	66
0.3	12	15	13.5	0.5	5.5
0.075	8	10	9	4.5	49.5
Pan	0	0	0	9	99
				**100**	**1100**

**Table 4:** Properties of coarse and fine aggregate.

Properties	Standard test	Test results	Standard requirement
L.A. abrasion (%)	ASTM C-131	21%	Below 30%
Flakiness index (%)	BS 182: part 3	4%	Below 20%
Elongation index (%)	BS 182: part 3	14.3%	Below 20%
Soundness (%)	BS 12: part 3	4.1%	Below 12%
Impact value (%)	BS 12: part 3	12.4%	Below 15%
Polished stone value (%)	BS 12: part 3	48.4%	Above 40%

**Table 5:** The physical properties of crumb rubber (CR).

Physical properties	Unit	Standard
Density,	1319 kg/m^3^	ASTM D1817
Young's modulus (*E*)	2600–2900 MPa	ASTM D1415
Tensile strength (*σ*_*t*_)	40–70 MPa	ASTM D 412
Elongation at break	25–50%	ASTM C1619
Melting point	200°C	ASTM D 1519

**Table 6:** Chemical components of CRM number 40 (ASTM D297).

Chemical components	Value
Acetone extract (%)	23.1
Rubber hydrocarbon (%)	46.6
Carbon black content (%)	25.08
Natural rubber content (%)	43.85
Ash content (%)	5.2
Particle size (*μ*)	425

**Table 7:** Illustration of Marshall characteristics (ASTM D 1559).

CR (%)	Stability (kN)	Flow (mm)	CDM (g/mL)∗	VIM (%)∗∗
0	10.4	3.5	2.53	3.16
6	10.9	3.2	2.40	3.30
8	11.2	3.0	2.38	4.19
10	11.71	2.80	2.30	4.45
12	12.1	2.10	2.12	5.12

*CDM: density of the compacted mix; **VIM: voids in the mix.

**Table 8:** Fatigue results and fatigue prediction equations.

CR (%)	*σ* (kPa)	*με*	*N*_*f*_ (cycles)	Fatigue module	*K*1	*K*2	*R*²
0	200	1188	15,230	*N*_*f*_ = 2.45 × 10^13^(1/*ε*)^0.322^	2.23 × 10^3^	1.45	0.89
	300	3400	3200	*N*_*f*_ = 2.361 × 10^12^(1/*ε*)^0.22^	2.45 × 10^3^	1.65	0.85
	400	9800	340	*N*_*f*_ = 2.70 × 10^10^(1/*ε*)^1.1^	1.99 × 10^10^	1.87	0.88

6	200	688	18990	*N*_*f*_ = 2.87 × 10^11^(1/*ε*)^1.111^	2.44 × 10^13^	1.34	0.94
	300	2450	4004	*N*_*f*_ = 3.56 × 10^12^(1/*ε*)^0.322^	2.88 × 10^11^	1.66	0.922
	400	6545	560	*N*_*f*_ = 3.87 × 10^12^(1/*ε*)^2.112^	2.09 × 10^13^	1.87	0.933

8	200	540	19500	*N*_*f*_ = 2.89 × 10^12^(1/*ε*)^1.312^	1.989 × 10^11^	1.97	0.94
	300	2200	5100	*N*_*f*_ = 3.55 × 10^12^(1/*ε*)^2.22^	2.344 × 10^11^	1.88	0.951
	400	4200	689	*N*_*f*_ = 2.99 × 10^12^(1/*ε*)^0.11^	2.11 × 10^10^	2.11	0.911

10	200	891	18590	*N*_*f*_ = 3.10 × 10^10^(1/*ε*)^0.23^	1.98 × 10^10^	2.88	0.944
	300	1750	4189	*N*_*f*_ = 2.78 × 10^10^(1/*ε*)^1.03^	2.93 × 10^11^	2.99	0.935
	400	6454	722	*N*_*f*_ = 3.11 × 10^11^(1/*ε*)^0.03^	2.78 × 10^10^	3.11	0.916

12	200	945	19888	*N*_*f*_ = 2.56 × 10^13^(1/*ε*)^1.3^	2.90 × 10^10^	2.88	0.899
	300	3550	4900	*N*_*f*_ = 2.88 × 10^13^(1/*ε*)^1.10^	2.95 × 10^11^	2.90	0.970
	400	7899	888	*N*_*f*_ = 4.23 × 10^13^(1/*ε*)^2.1^	3.11 × 10^12^	2.89	0.99

**Table 9:** Regression coefficient between fatigue strain and creep strain and stiffness using SPSS analysis.

Stresses (kPa)	Regression coefficient (*R*²)	
	Stiffness	Creep
200	0.95	0.71
300	0.89	0.67
400	0.93	0.68

## References

[1] Hamed FKM (2010). *Evaluation of fatigue resistance for modified asphalt concrete mixture based on dissipate energy concept [Ph.D. thesis]*.

[2] Mahrez A (2008). *Properties and performance of stone mastic asphalt reinforced with glass fibre [Ph.D. thesis]*.

[3] Adedimila AS, Kennedy TW (1976). Repeated-load indirect tensile fatigue characteristics of asphalt mixture. *Transportation Research Record*.

[4] Mashaan NS, Ali A, H A, Koting S, Karim MR (2013). Dynamic properties and fatigue life of stone mastic asphalt mixtures reinforced with waste tyre rubber. *Advances in Materials Science and Engineering*.

[5] Brown ER, Hemant M (1993). Evaluation of laboratory properties of SMA mixture. *NCATR Report*.

[6] Ratnasamy M, Bujang BK (2006). Laboratory diameteral fatigue performance of SMA with cellulose oil palm fiber. *American Journal of Applied Sciences*.

[7] Mashaan NS, Ali AH, Koting S, Karim MR (2013). Performance evaluation of crumb rubber modified stone mastic asphalt pavement in Malaysia. *Advances in Materials Science and Engineering*.

[8] Mashaan NS, Ali AH, Karim MR, Mahrez A (2014). A Review of using crumb rubber in reinforcement of asphalt pavement. *The Scientific World Journal*.

[9] Mashaan NS, Karim MR (2013). Evaluation of permanent deformation of CRM-reinforced-SMA and its correlation with dynamic stiffness and dynamic creep. *The Scientific World Journal*.

[10] Mashaan NS, Karim MR (2013). Investigating the rheological properties of crumb rubber modified bitumen and its correlation with temperature susceptibility. *Materials Research*.

[11] Raad L, Saboundjian S (1998). Fatigue behavior of rubber-modified pavements. *Transportation Research Record*.

[12] Soleymani HR, Zhai H, Bahia H (2004). Role of modified binders in rheology and damage resistance behavior of asphalt mixtures. *Transportation Research Record*.

[13] McGennis RB (1995). Evaluation of physical properties of fine crumb rubber-modified asphalt binders. *Transportation Research Record*.

[14] Billiter TC, Davison RR, Glover CJ, Bullin JA (1997). Physical properties of asphalt-rubber binder. *Petroleum Science and Technology*.

[15] Roberts FL, Kandhal PS, Brown ER, Lee DY, Kennedy TW (1991). *Hot Mix Asphalt Materials, Mixture Design and Construction*.

[16] Barksdale RD (1978). Partical application of fatigue and rutting tests on bituminous base mixes. *Asphalt Paving Technol*.

[17] Raithby KD, Ramshaw JT (1972). Effects of secondary compaction on the fatigue performance of a hot-rolled asphalt.

[18] Read JM, Collop AC (1997). Partical fatigue characterisation of bituminous paving mixture. *Journal of the Association of Asphalt Paving Technologist*.

[19] Raithby KD, Sterling AB (1970). The effect of rest period on the fatigue performance of a hot-rolled asphalt under reversed axial loading. *Journal of the Association of Asphalt Paving Technologist*.

[20] Bonnaure FP, Gravois A, Udron J (1980). A new method for predicting the fatigue life characteristics of bituminous mixes. *Journal of the Association of Asphalt Paving Technologist*.

[21] Raithby KD, Sterling AB (1972). Some effect of loading history on the fatigue performance of rolled asphalt.

[22] Van DW, Visser W (1977). The energy approach to fatigue for pavement design. *Journal of the Association of Asphalt Paving Technologist*.

[23] Pell PS, Cooper KE (1975). The effect of testing and mix variables on the fatigue performance of bituminous materials. *Journal of the Association of Asphalt Paving Technologist*.

[24] Baladi G (1989). Fatigue life and permanent deformation characteristics of asphalt concrete mixes. *Transportation Research Record*.

[25] Goddard RTN, Powell WD, Applegate MW (1978). Fatigue resistance of dense bitumen macadam: the effect of mixture variables and temperature. *TRRL Suppl. Report*.

[26] Epps J, Monismith CL (1971). Fatigue of asphalt concrete mixture-summary of existing information. *Special Technical Report*.

[27] Nejad FM, Aflaki E, Mohammadi MA (2010). Fatigue behavior of SMA and HMA mixtures. *Construction and Building Materials*.

[28] Arabani M, Mirabdolazimi SM, Sasani AR (2010). The effect of waste tire thread mesh on the dynamic behaviour of asphalt mixtures. *Construction and Building Materials*.

